# Multi-dimensional dynamic visualization of spatiotemporal data for tactical analysis in table tennis

**DOI:** 10.3389/fbioe.2026.1876984

**Published:** 2026-07-23

**Authors:** Jianwei Guo, Qingyun Huang, Lei Gao, Xingdong Zhou, Lei Qian, Jing Sun, Dandan Xiao

**Affiliations:** 1 School of Athletic Performance, Shanghai University of Sport, Shanghai, China; 2 School of Artificial Intelligence and Computer Science,North China University of Technology, Beijing, China; 3 Beijing Chuangxin Zhijiu Technology Co., Ltd, Beijing, China; 4 The Faculty of Sports Science, Ningbo University, Ningbo, China; 5 Physical Education School, Xian Technological University, Xian, China; 6 College of Sports and Health, Shandong Sport University, Jinan, China

**Keywords:** cluster analysis, parallel coordinates, spatiotemporal cube, spatiotemporal data, table tennis, visual analytics

## Abstract

**Introduction:**

Table tennis is a high-speed racket sport that generates complex technical and tactical data, including stroke sequences, landing-point distributions, shot techniques, player names, and point outcomes. Traditional statistical charts and two-dimensional visualizations are often limited in representing multidimensional categorical relationships and the dynamic evolution of landing patterns across rallies. This study therefore aims to develop a visual analytics framework for exploratory post-match technical and tactical diagnosis in table tennis.

**Methods:**

A multimodal visual analytics framework was developed by integrating a cluster-enhanced parallel coordinates module with a stroke-sequence-oriented spatiotemporal cube module. In the parallel coordinates module, K-Modes clustering was used to aggregate categorical stroke-event records into representative tactical profiles, thereby reducing line occlusion and revealing co-occurrence patterns among player name, stroke number, stroke position, landing position, shot technique, and point outcome. In the spatiotemporal cube module, landing points within each rally were modeled as discrete landing-point sequences, and sub-trajectory clustering was applied to identify recurring landing-path patterns and the evolution of landing hotspots across stroke sequences. Two independent match cases with reliability-checked annotations were used to evaluate the two modules according to their respective analytical objectives. In addition, five experts assessed the system using a seven-point Likert scale.

**Results:**

The results indicated that the cluster-enhanced parallel coordinates module improved the readability of multidimensional categorical data and supported the identification and interpretation of player-specific tactical profiles. The spatiotemporal cube module revealed recurring landing-path patterns and stroke-sequence-based changes in landing-point distributions. The expert evaluation further supported the analytical usefulness and interpretability of the proposed framework for post-match technical and tactical analysis.

**Discussion:**

By combining categorical tactical-pattern analysis with spatiotemporal landing-sequence analysis, the proposed framework provides a domain-adapted approach for examining both multidimensional technical-tactical relationships and the dynamic evolution of landing patterns in table tennis. The framework supports exploratory tactical diagnosis and offers methodological implications for the visualization and analysis of spatiotemporal data in racket sports.

## 1 Introduction

Data visualization facilitates the extraction of pivotal insights and the representation of multidimensional attributes by transforming abstract datasets into intuitive visual structures (Keim et al., 2008; Kehrer and Hauser, 2012). With recent technological advancements, visual analytics has been extensively integrated into sports competition analysis to graphically characterize athletes’ technical and tactical behaviors (Perin et al., 2018; Du and Yuan, 2021). The core objective of this field is to empower athletes and coaches to optimize performance and enhance win rates through the rigorous analysis of behavioral data. In competitive sports, such data typically encompasses individual physiological metrics and interactive behavioral patterns, characterized by high-dimensional spatiotemporal, descriptive, and statistical properties (Thornton et al., 2019; Du and Yuan, 2021).

In the contemporary table tennis landscape, the overall elevation of competitive standards has profoundly transformed match dynamics (Fuchs et al., 2018). The narrowing gap in skill levels and the accelerating pace of play mean that victory often hinges on the meticulous execution of each rally and the precision of tactical transitions (Fuchs et al., 2018; Lai et al., 2018). As a high-speed, elite-level sport, table tennis generates vast volumes of multidimensional spatiotemporal data, including stroke trajectories, landing point distributions, and player movement paths (Wu et al., 2018). This data inherently possesses complex temporal logic (the sequential order of strokes) and rich tactical attributes (the specific technique employed and the resulting point won/lost) (Wang et al., 2021). While these datasets offer a crucial foundation for uncovering tactical patterns, they pose significant challenges to traditional analysis methods that rely heavily on basic statistics or subjective experience. Recent studies highlight the limitations of these conventional methods; for instance, relying solely on subjective evaluations or isolated outcomes often fails to capture the multifaceted complexities of tactical performance (Gan et al., 2025). Conventional statistical charts and textual descriptions often fail to capture the deep correlations between multidimensional attributes or the dynamic evolution of tactics, leading to “information gaps” and cognitive overload when processing complex match data (Perin et al., 2018).

Against this backdrop, spatiotemporal visualization offers unique analytical value (Andrienko et al., 2013; Wu et al., 2022). By mapping data across temporal and spatial dimensions, this approach uncovers latent evolutionary patterns and intrinsic connections that are otherwise obscured (Wu et al., 2022). Specifically, spatiotemporal techniques can graphically highlight key technical features across varying temporal granularities, facilitating a deeper understanding of match dynamics. Although researchers have employed diverse tools—such as scatter plots for landing point analysis and heatmaps for hitting hotspots (Boonim, 2023; Chen et al., 2023)—traditional sports visualization often remains restricted to one-dimensional metrics (e.g., scores) or static representations (Straub and Klein-Soetebier, 2017). These methods struggle to resolve the complexities of modern table tennis, where the rapid, intuitive, and in-depth analysis of high-dimensional sequences is now a critical requirement (Wang et al., 2023).

Despite its burgeoning importance, visualization research in table tennis remains constrained by narrow analytical scopes and technical bottlenecks, most notably visual clutter and line occlusion in complex datasets (Artero et al., 2004; Ellis and Dix, 2007; Perin et al., 2018; Wu et al., 2018). To address these limitations, this study proposes an integrated multimodal visualization framework designed to reduce perceptual confusion and enhance the interpretability of table tennis tactical analysis. The primary contributions of this research are threefold. First, we present an enhanced parallel coordinates visualization method based on K-Modes clustering, which is suitable for categorical tactical variables such as stroke number, landing position, and shot technique. This method aggregates categorical stroke-event records into representative tactical patterns, thereby supporting the identification of athletes’ tactical preferences and the associations between technical execution and point outcomes. Second, we develop a stroke-sequence-oriented spatiotemporal cube visualization method that conceptualizes rallies as discrete landing-point sequences. By representing rally progression across successive stroke numbers and incorporating trajectory-based aggregation, this method provides a three-dimensional perspective on the dynamic evolution of landing distributions and tactical routines. Finally, these methods are integrated into a table tennis visual analytics system, which supports interactive exploration of player-specific tactical characteristics and provides visual evidence for post-match technical and tactical diagnosis.

## 2 Related work

### 2.1 Visual and performance analysis in table tennis

Visual analytics has been extensively integrated into sports competition analysis to graphically characterize athletes’ technical and tactical behaviors (Keim et al., 2008; Perin et al., 2018; Du and Yuan, 2021). While foundational research initially focused on team sports to track player movements, analyze passing dynamics (Legg et al., 2012; Andrienko et al., 2013), and augment live game-viewing experiences, racket sports present unique challenges due to their high-speed nature and the generation of complex, multidimensional spatiotemporal data. Previous frameworks, such as TenniVis for tennis (Polk et al., 2014), CourtTime for tennis match visual analytics (Polk et al., 2019), Shuttlespace for badminton (Ye et al., 2020), and TacticFlow for visualizing ever-changing tactical progressions (Wu et al., 2022), have pioneered the visual analysis of stroke sequences and court coverage. These studies provide important methodological references for racket-sport visualization; however, their analytical contexts differ from table tennis in terms of court structure, rally rhythm, and stroke-sequence logic.

In the realm of sports science, notational analysis has long been established as a primary method for dissecting table tennis tactics (Malagoli Lanzoni et al., 2014). Comprehensive systematic reviews (Fuchs et al., 2018; Zhang et al., 2018) and empirical studies (Jin and Ma, 2026; Chen et al., 2025) have extensively analyzed stroke attributes—such as technique, landing position and stroke sequences—to evaluate elite players’ performance. Furthermore, research has specifically highlighted the critical role of spatial distributions and playing zones in determining match outcomes (Fuchs et al., 2018). However, these traditional sports science methodologies rely heavily on static statistical charts and discrete performance indicators. As the complexity of match data grows, these conventional approaches often lead to cognitive overload and fail to capture the deep, dynamic relationships between multidimensional attributes (Mining, 2006; Ellis and Dix, 2007).

To bridge this gap, the visual analytics community has made significant advancements. Systems such as iTTVis (Wu et al., 2018) laid the groundwork for interactive visualization of table tennis match data and ball trajectories, Tac-Simur introduced a tactic-based simulative visual analytics framework for modeling and exploring table tennis tactical processes (Wang et al., 2019), and Tac-Miner focused on mining sequential rally transitions and tactical patterns from match data (Wang et al., 2021). These studies represent important advances in table tennis visual analytics, but they address different analytical tasks from the present study. iTTVis emphasizes interactive exploration of ball trajectories and match data (Wu et al., 2018), Tac-Simur focuses on tactic simulation and tactical process exploration (Wang et al., 2019), and Tac-Miner emphasizes tactic mining from match data (Wang et al., 2021). In contrast, the present study focuses on two specific visualization problems in post-match table tennis technical and tactical analysis: reducing visual clutter and line occlusion in multidimensional categorical stroke-event data, and representing the dynamic evolution of landing point distributions across stroke sequences. By combining K-Modes-based aggregation in parallel coordinates with stroke-sequence-oriented spatiotemporal visualization, this study provides a complementary visual analytics framework for identifying representative tactical patterns and supporting technical and tactical diagnosis in table tennis.

### 2.2 Multidimensional data visualization and parallel coordinates

Analyzing table tennis tactical behavior requires the simultaneous consideration of multiple categorical attributes, including stroke number, stroke position, landing position, shot technique, and point won/lost. Parallel coordinates are effective for displaying relationships among multidimensional variables within a unified visual structure (Inselberg, 2008; Heinrich and Weiskopf, 2013). This makes them suitable for exploring the associations among technical and tactical attributes that are difficult to observe in separate statistical charts. However, when each stroke event is directly represented as an individual polyline, traditional parallel coordinate plots may suffer from severe visual clutter and line occlusion (Keim, 2002; Artero et al., 2004; Ellis and Dix, 2007). In table tennis tactical analysis, this problem becomes more pronounced because many stroke events contain repeated or similar categorical combinations, such as the same stroke number, landing position, and shot technique. As a result, raw event-level plotting may obscure representative tactical patterns and reduce the readability of multidimensional visual analysis.

To address this problem, this study combines the characteristics of table tennis technical and tactical analysis with K-Modes clustering to construct a categorical tactical-pattern aggregation approach. K-Modes is suitable for nominal categorical variables because it measures similarity through category matching and represents each cluster by the dominant category of each attribute (Huang, 1998). In the context of table tennis, this allows stroke-event records with similar combinations of stroke number, stroke position, landing position, and shot technique to be grouped into representative tactical profiles. These profiles can then be visualized in parallel coordinates to reduce repeated line crossings and support the interpretation of dominant technical and tactical patterns. In the proposed framework, K-Modes-based aggregation is used as a domain-adapted visual analysis strategy rather than as a universal classification of table tennis tactics. In this way, the parallel coordinates module improves the readability of multidimensional categorical tactical data and supports exploratory interpretation of the co-occurrence relationships among player name, stroke-event attributes, and point outcome.

### 2.3 Spatiotemporal sequence visualization

In addition to multidimensional categorical relationships, table tennis tactics also have a strong spatiotemporal structure (Fuchs et al., 2018; Zhang et al., 2018). The tactical meaning of a stroke depends not only on its shot technique and landing position, but also on its position within the stroke sequence. For example, the same landing position may have different tactical implications when it appears during the serve, receive, third stroke, fourth stroke, fifth stroke, or rally phase. Therefore, visualizing the evolution of landing point distributions across stroke sequences is important for understanding tactical progression in table tennis. Spatiotemporal visualization provides an effective way to represent spatial changes over time or sequence. The Space-Time Cube is a classic model for integrating spatial position and temporal progression into a unified three-dimensional representation (Kraak, 2003; Gatalsky et al., 2004; Bach et al., 2014). By mapping temporal or sequential information to the vertical dimension, this model allows analysts to observe how spatial patterns evolve across a continuous process. In sports visual analytics, related systems such as CourtTime (Polk et al., 2019) and TacticFlow (Wu et al., 2022) have also shown that temporal structure is important for interpreting shot patterns and tactical evolution in racket sports. However, for table tennis tactical analysis, the temporal dimension is often more meaningfully represented by stroke sequence than by generic clock time, because coaches and analysts usually interpret rally development through serve, receive, third stroke, fourth stroke, fifth stroke, and rally.

For stroke-sequence-based table tennis data, landing points within a rally can be represented as discrete spatiotemporal sequences. In this context, sub-trajectory clustering provides a useful way to extract recurring local patterns from landing-point trajectories rather than treating each rally as an undifferentiated whole. This is particularly relevant to table tennis technical and tactical analysis because tactical routines are often reflected in short sequential combinations, such as serve-to-third-stroke placement, receive-to-fourth-stroke transition, or landing-position shifts before entering the rally phase. By grouping similar sub-trajectories, analysts can identify representative landing-path patterns and examine how spatial placement changes across different stroke sequences.

Based on these considerations, the present study combines the characteristics of table tennis technical and tactical analysis with a stroke-sequence-oriented spatiotemporal visualization model and sub-trajectory clustering. This design aims to represent the dynamic evolution of landing point distributions across rally progression and to extract recurring landing-path patterns from sequential stroke data. In this way, the spatiotemporal visualization module provides a methodological basis for analyzing how spatial placement evolves within table-tennis-specific stroke sequences, while complementing the multidimensional analysis of categorical stroke-event relationships.

## 3 Research methodology

### 3.1 Data acquisition and description

**FIGURE 1:**
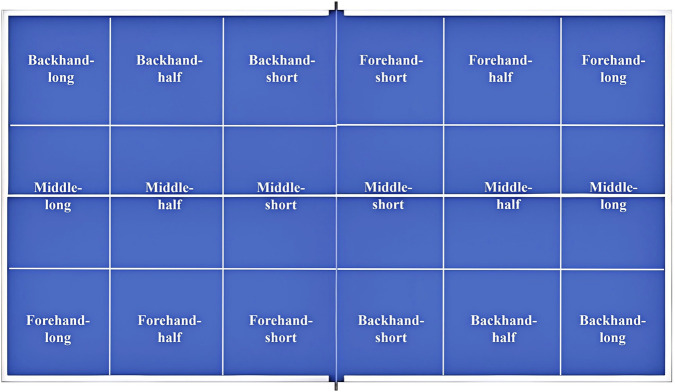
Diagram of table tennis landing zone classification.

**FIGURE 2:**
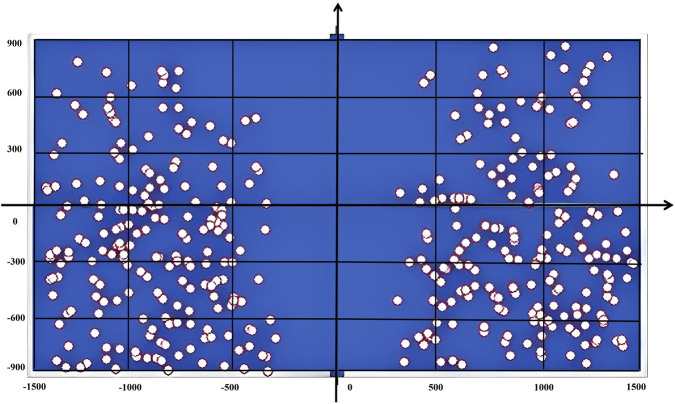
Ball Landing Point Coordinate System. This landing point is solely for visualization in the table tennis spatiotemporal cube. The landing point coordinates are (Landing Position X,Landing Position Y).

Two independent match cases were used to evaluate the proposed visual analytics framework according to the respective analytical objectives of the two visualization modules. Both cases were selected from finals of two different high-level international men’s table tennis events held in 2026, and all participating athletes were top-level male table tennis players. Match Case 1 was used to validate the cluster-enhanced parallel coordinates module, which focuses on multidimensional categorical stroke-event relationships. This match ended with a final score of four to three and contained 150 rallies, providing complete categorical stroke-event annotations for subsequent K-Modes-based aggregation and parallel coordinates visualization. Match Case 2 was used to validate the stroke-sequence-oriented spatiotemporal cube module, which focuses on landing-point evolution and recurring landing-path patterns across stroke sequences. This match ended with a final score of four to two and contained 109 rallies, from which 487 valid landing-point records were obtained for landing-point sequence construction, hotspot distribution analysis, and sub-trajectory clustering. Raw match footage was annotated using specialized video analysis software according to the predefined indicator system. For categorical stroke-event annotation, two trained annotators independently completed the coding process, and inconsistent cases were reviewed and resolved together with a third researcher. For landing-point coordinate annotation, reliability was assessed using the intraclass correlation coefficient (ICC). This module-specific validation design allows the two analytical pathways to be examined according to their different data requirements while maintaining annotation reliability. The core attributes, detailed in Table 1, collectively characterize individual stroke events and landing-point information. Specifically, Player Name identifies the competitors, while Stroke Number denotes the sequential order of shots within a rally. The sequence includes Serve, Receive, Third Stroke, Fourth Stroke, Fifth Stroke, Sixth Stroke, and Rally. Spatial metrics are defined by Stroke Position and Landing Position, both mapped to a nine-zone grid (Figure 1). For precise localization, Landing Position X/Y records the spatial coordinates within a Cartesian system (Figure 2). Shot Techniques classify specific maneuvers, and Point Won/Lost documents the final outcome of the rally.

**TABLE 1:** Data attributes and specifications.

Indicator names	Value range
Player name	{Player A, player B}
Stroke number	{Serve, Receive,Third Stroke, Fourth Stroke, Fifth stroke, sixth Stroke, Rally}
Stroke position	{Forehand-long,Forehand-half,Forehand-short,Middle-long, middle-half,middle-short,backhand-long,backhand-half,backhand-short}
Landing position	{Forehand-long,Forehand-half,Forehand-short,Middle-long, middle-half,middle-short, backhand-long,backhand-half, backhand-short}
Landing position X	[-1500,1500]
Landing position Y	[-900,900]
Shot techniques	{Forehand Loop, Backhand Loop, Side Loop, Twist,Flick, Short push, Long push}
Point won/Lost	{Won,Lost}

### 3.2 Research framework

To mitigate challenges such as visual clutter, information overlap, and the inadequate representation of dynamic features in high-dimensional table tennis data, this study developed an integrated analytical framework. The framework encompasses data preprocessing, pattern mining, and multidimensional visualization. Utilizing multidimensional stroke event data from elite international competitions, the framework is structured around two parallel analytical pathways:

1. K-Modes-Based Parallel Coordinates Visualization: This pathway focuses on the visual analysis of multidimensional categorical stroke-event attributes, including player name, stroke number, stroke position, landing position, shot technique, and point won/lost. To reduce visual clutter and line occlusion in raw parallel coordinates, K-Modes clustering is employed to aggregate stroke-event records with similar categorical combinations into representative tactical profiles. Each profile is characterized by the dominant category of each variable, making the clustered results interpretable in relation to table tennis technical and tactical analysis. These aggregated tactical profiles are then visualized through parallel coordinates to support the interpretation of co-occurrence patterns among player name, stroke-event attributes, and point outcomes.
2. Stroke-Sequence-Oriented Spatiotemporal Cube Visualization: This pathway addresses the spatiotemporal dynamics of landing-point sequences by treating the landing points within each rally as discrete spatiotemporal sequences. In table tennis tactical analysis, the temporal dimension is more meaningfully represented by stroke sequence than by generic clock time. Therefore, the framework organizes landing-point data according to stroke number, such as serve, receive, third stroke, fourth stroke, fifth stroke, and rally. Based on the Space-Time Cube model, the horizontal dimensions represent landing-point coordinates, while the vertical dimension represents the stroke-sequence progression. By incorporating trajectory segmentation and sub-trajectory clustering, the framework extracts recurring landing-path patterns and hotspot distributions across successive stroke numbers. This pathway enables analysts to observe how landing point distributions evolve during rally progression and to identify repeated spatial routines embedded in table tennis stroke sequences.

Finally, these two analytical pathways are synthesized into a unified visualization analysis system based on the Model-View-Controller (MVC) architecture. The K-Modes-based parallel coordinates pathway supports the abstraction and interpretation of categorical tactical patterns, while the stroke-sequence-oriented spatiotemporal cube pathway supports the analysis of landing-distribution evolution and recurring landing-path patterns. Through interactive, multi-perspective exploration, the system enables analysts to examine technical and tactical characteristics from both multidimensional attribute and spatiotemporal sequence perspectives, thereby providing visual evidence for post-match table tennis technical and tactical diagnosis.

**FIGURE 3:**
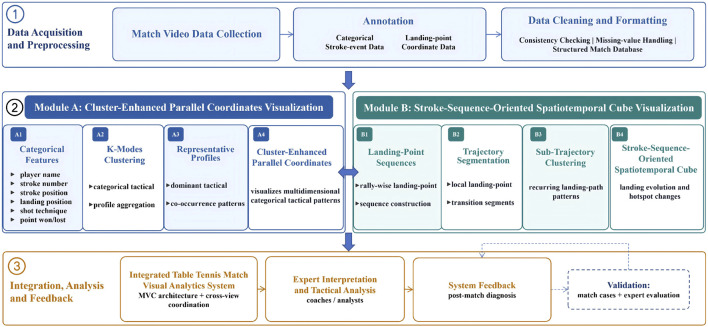
Overall workflow of the proposed visual analytics framework.

To further clarify the overall model construction process, Figure 3 presents the complete workflow of the proposed visual analytics framework. The workflow begins with raw match video data collection, followed by manual or software-assisted annotation, data cleaning, consistency checking, and structured data formatting. After preprocessing, the framework proceeds along two complementary analytical pathways: a K-Modes-based categorical stroke-event pathway for cluster-enhanced parallel coordinates visualization and a landing-point sequence pathway for sub-trajectory clustering and stroke-sequence-oriented spatiotemporal cube visualization. The two pathways are then integrated into the table tennis match visual analytics system through cross-view coordination, supporting expert interpretation, tactical analysis, and system feedback.

## 4 Research results and implementation

### 4.1 Cluster-enhanced parallel coordinates visualization

Parallel coordinates are highly effective for uncovering trends and interrelationships in high-dimensional spatiotemporal data (Inselberg, 2008; Heinrich and Weiskopf, 2013). However, traditional parallel coordinate plots often suffer from visual clutter and “overplotting” when processing extensive match datasets (Keim, 2002; Artero et al., 2004; Ellis and Dix, 2007). To address this, we developed a cluster-enhanced parallel coordinates method designed to reveal complex tactical associations, specifically, the correlations between player name, stroke number, stroke position, technique, and the resulting point won/lost.

The workflow consists of four primary stages: (1) data initialization and structural preprocessing; (2) categorical attribute standardization; (3) tactical-pattern aggregation using K-Modes clustering; and (4) multidimensional visualization and tactical interpretation. Different from numerical feature-based visualization pipelines, the present module is designed for nominal tactical attributes. Therefore, the preprocessing stage focuses on category consistency, missing-value checking, and structural organization. The resulting aggregated profiles are then visualized through parallel coordinates to reduce visual clutter and support the interpretation of representative technical and tactical patterns. To validate the cluster-enhanced parallel coordinates module, Match Case 1 was used as the categorical stroke-event analysis case. This match was selected from the final of a high-level international men’s table tennis event held in 2026, ended with a final score of four to three, and contained 150 rallies. Tactical indicators were annotated for each rally, resulting in 1,350 coded items per annotator. The two annotators reached an overall agreement rate of 98.74%, and inconsistent annotations were reviewed and resolved together with a third researcher before the corrected dataset was used for K-Modes-based aggregation and visualization.

#### 4.1.1 Data preprocessing and categorical coding

Before clustering and visualization, the stroke-event records were checked and organized according to the predefined coding protocol. Each stroke event was represented by multiple categorical variables, including player name, stroke number, stroke position, landing position, shot technique, and point won/lost. These variables describe the player involved in the stroke event, the sequential position of the stroke within the rally, the spatial and technical characteristics of the stroke, and the corresponding point outcome.

All category labels were reviewed to ensure coding consistency. Inconsistent expressions, spelling variations, and ambiguous category assignments were corrected before clustering. Records with uncertain annotations were checked against the original match record and resolved according to the unified coding protocol. The corrected categorical dataset was then used as the input for K-Modes clustering.

Let the categorical stroke-event dataset be denoted as $D=\left\{x_{1}{,x}_{2}{,...,x}_{n}\right\}$ , where n represents the number of stroke-event records. Each record xᵢ is represented by m categorical variables, namely, $x_{i}=\left(x_{i1},x_{i2},...,x_{im}\right)$ . In this study, these variables include player name, stroke number, stroke position, landing position, shot technique, and point won/lost. Therefore, each record represents a complete categorical description of one stroke-event pattern in the analyzed match.

#### 4.1.2 Tactical pattern extraction via K-Modes clustering and K-Value selection

The purpose of clustering in this section is to reduce visual clutter in parallel coordinates and to extract representative categorical tactical profiles from the original stroke-event records. Rather than displaying every individual stroke event as an independent line, K-Modes clustering groups records with similar categorical combinations into a smaller number of representative profiles. This design is consistent with the analytical needs of table tennis technical and tactical diagnosis, where coaches and analysts are often more interested in typical combinations of player name, stroke number, stroke position, landing position, shot technique, and point won/lost than in isolated event records.

For categorical variables, the dissimilarity between two records is determined by whether their corresponding attribute values are identical. Let q_l_ denote the mode of cluster C_l_. The dissimilarity between a stroke-event record x_i_ and the cluster mode q_l_ is defined as:

$$d\left(x_{i}{,q}_{l}\right)=\Sigma_{j}\;\delta \left(x_{\text{ij}}{,q}_{\text{1jj}}\right),j=1,...,m$$

where delta (x_ij_, q_lj_) = 0 if x_ij_ = q_lj_, and delta (x_ij_, q_lj_) = 1 if x_ij_ ≠ q_lj_. In other words, the categorical dissimilarity is calculated by counting the number of mismatched attributes between a stroke-event record and a cluster mode.

The objective of K-Modes clustering is to minimize the total within-cluster categorical dissimilarity:

$$J=\Sigma_{i}{\;\Sigma }_{l}\;u_{\text{il}}\;d\left(x_{i}{,q}_{l}\right),i=1,...,n;\;l=1,...,K$$

where K is the number of clusters, and uil indicates whether stroke event x_i_ belongs to cluster C_l_. If xi belongs to C_l_, then u_il_ = 1; otherwise, u_il_ = 0. During the iterative process, each stroke-event record is assigned to the nearest cluster mode based on categorical dissimilarity. The mode of each cluster is then updated by selecting the most frequent category of each attribute within that cluster:

$$q_{lj}=mode\left\{x_{ij}\;|\;x_{i}{\in C}_{l}\right\}$$

As a result, each cluster is represented by a tactical mode composed of the dominant categories of player name, stroke number, stroke position, landing position, shot technique, and point won/lost. These modes can be interpreted as representative categorical tactical profiles in the analyzed match.

To determine an appropriate number of clusters, K values from 2 to 8 were tested. The selection of K jointly considered clustering cost, silhouette score based on categorical dissimilarity, convergence behavior, and visual interpretability. The clustering cost decreased as K increased, indicating reduced within-cluster categorical dissimilarity. However, the decrease became limited after K = 7; specifically, the cost decreased from 259 at K = 7 to 255 at K = 8, while the silhouette score dropped from 0.2135 to 0.1535. Therefore, K = 7 provided a relatively better balance among aggregation compactness, categorical separation, convergence behavior, and visual readability, and was selected as the final configuration for the K-Modes-based aggregation.

It should be emphasized that K = 7 does not indicate that table tennis tactics can be universally divided into seven fixed categories. Rather, it means that the categorical stroke-event records in this match were aggregated into seven case-specific representative tactical profiles. These profiles are used as visual abstraction units to reduce line occlusion in parallel coordinates and to support exploratory interpretation of co-occurrence patterns among player name, stroke-event attributes, and point outcomes.

#### 4.1.3 Visualization implementation and configuration

After K-Modes clustering, the representative tactical profiles were mapped into the parallel coordinates system. The frontend visualization was implemented using the ECharts library. A unified global coordinate framework was established, and the main tactical dimensions, including player name, stroke number, stroke position, landing position, shot technique, and point won/lost, were bound to individual parallel axes.

The visualization was configured to present both the original stroke-event distribution and the aggregated tactical profiles. The raw event records were displayed as a background layer to preserve the overall data distribution, while the K-Modes cluster modes were rendered as foreground representative lines. Each foreground line corresponds to one tactical profile and shows the dominant categorical combination within that cluster. This layered design reduces the interference caused by dense line overlap while retaining the connection between the original records and the aggregated tactical patterns. The detailed workflow of the cluster-enhanced parallel coordinates visualization is shown in Algorithm 1.

Algorithm 1 **Cluster-Enhanced Parallel Coordinates Visualization based on K-Modes aggregation.**

 Input: categorical stroke-event dataset D, clustering variables A, and cluster number K.
 Output: cluster-enhanced parallel coordinates visualization.
 1. Initialize the global parallel coordinate framework.
 2. Bind tactical dimensions to parallel axes, including player name, stroke number, stroke position, landing position, shot technique, and point won/lost.
 3. Select the clustering variables A = {player name, stroke number, stroke position, landing position, shot technique, point won/lost}.
 4. Check categorical labels and construct the categorical stroke-event matrix D_A.
 5. Apply K-Modes clustering to D_A and obtain K representative tactical profiles.
 6. For each cluster C_l_:
  6.1. Identify the dominant category of each variable and generate the cluster mode q_l_.
  6.2. Convert the cluster mode into a representative polyline in the parallel coordinates system.
  6.3. Assign visual styles according to cluster identity and tactical profile characteristics.
 7. Render raw event records as a background layer when necessary.
 8. Render K-Modes tactical profiles as foreground representative lines.
 9. Support interactive filtering, brushing, and profile inspection.

#### 4.1.4 Parallel coordinates visualization results and tactical interpretation

**FIGURE 4:**
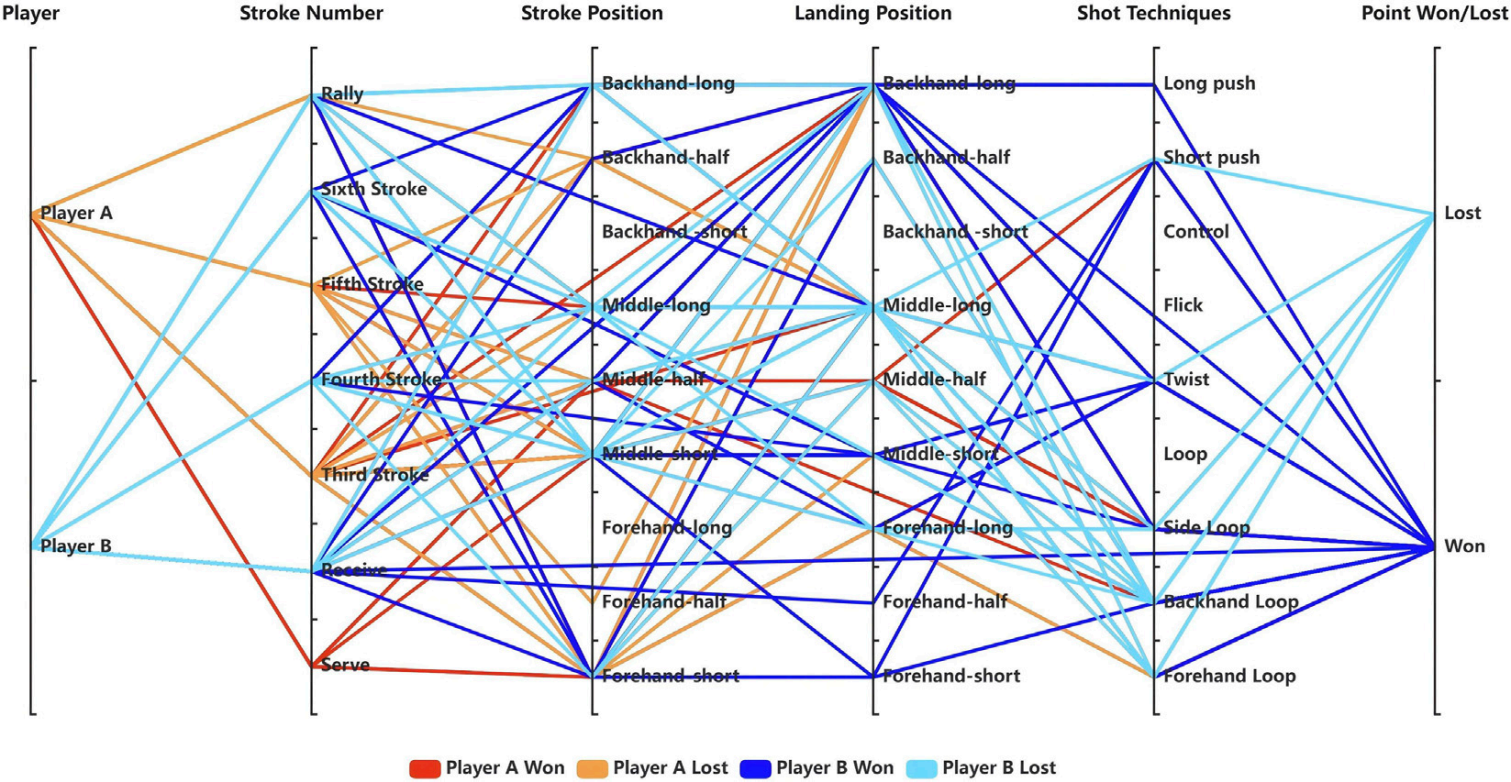
Original event-level parallel coordinates visualization of Match Case 1.

**FIGURE 5:**
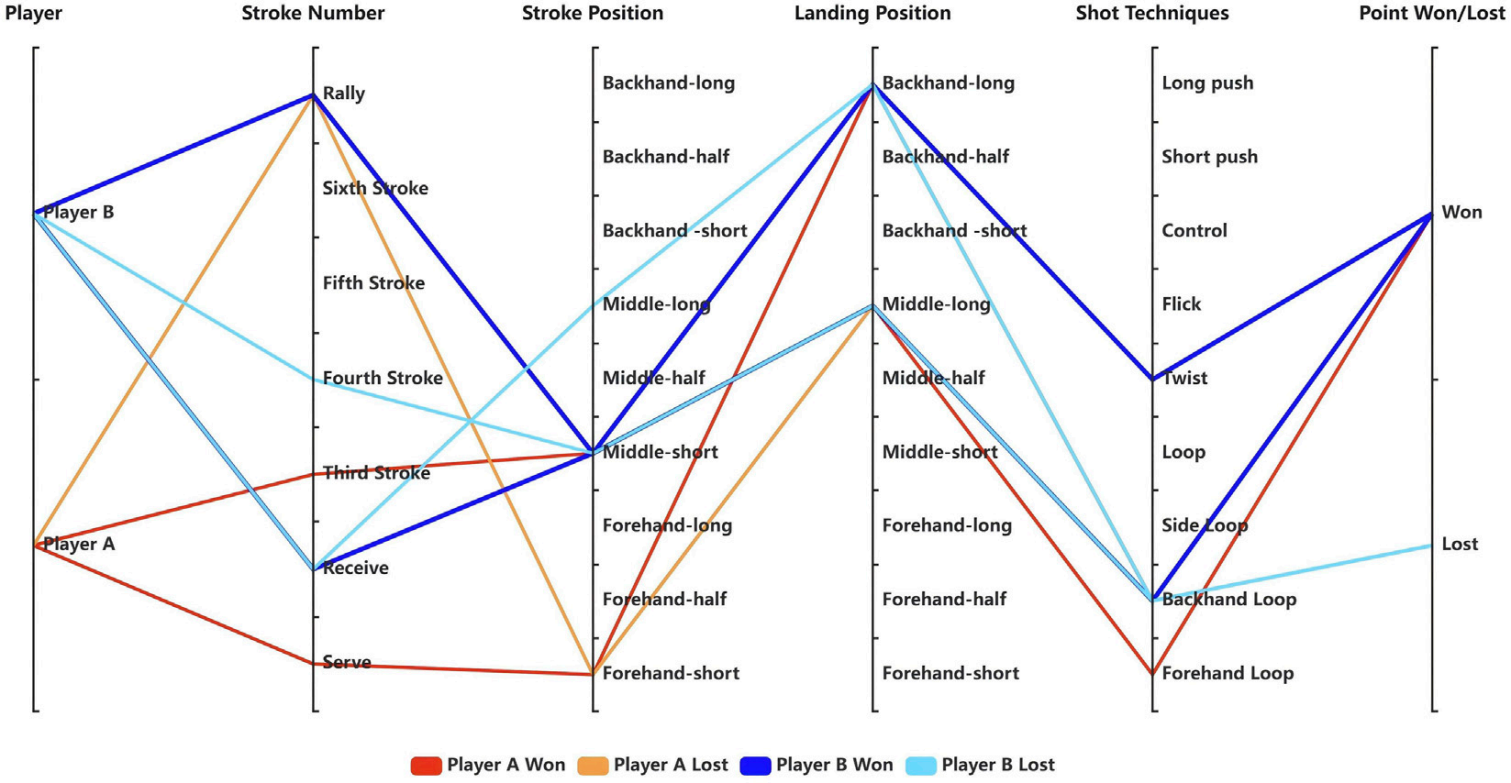
K-Modes-aggregated parallel coordinates visualization of Match Case 1.

The parallel coordinates visualization results are shown in Figures 4, 5. Figure 4 presents the original event-level parallel coordinates visualization, in which each stroke-event record is represented as an individual polyline. Although this view preserves the complete distribution of the match records, it also produces dense line crossing and visual overlap across categorical axes, especially in stroke number, stroke position, landing position, and shot technique. This makes it difficult to directly identify representative technical and tactical patterns from the raw visualization.

After K-Modes-based aggregation, the visualization becomes clearer because repeated or similar categorical combinations are summarized into representative tactical profiles. As shown in Figure 5, the aggregated view reduces redundant line crossings and clarifies the relationships among player name, stroke number, stroke position, landing position, shot technique, and point won/lost. Compared with the raw event-level display, the cluster-enhanced view allows analysts to focus on dominant technical and tactical combinations rather than isolated stroke events.

**FIGURE 6:**
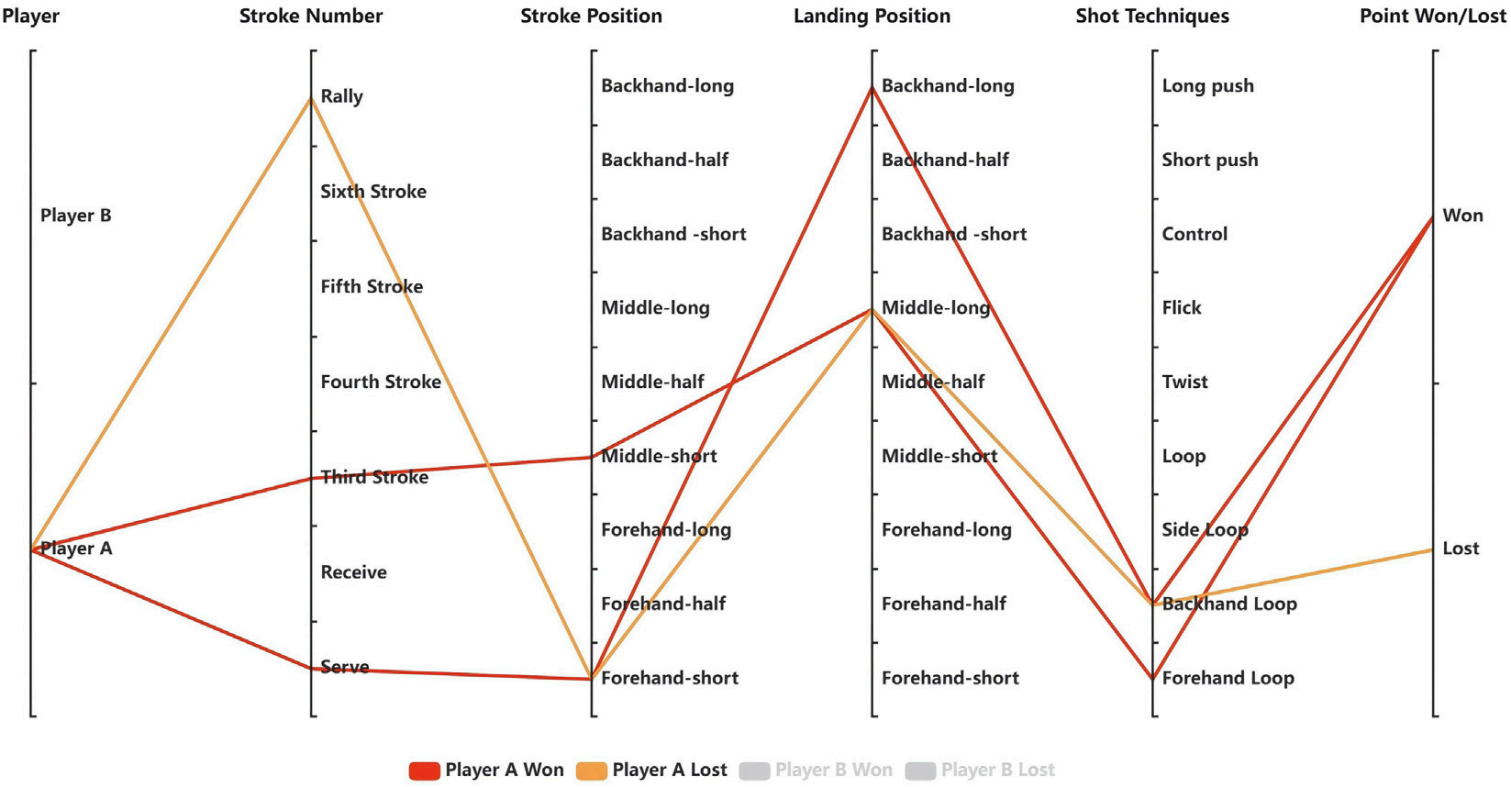
Player A tactical profiles derived from K-Modes aggregation.

**FIGURE 7:**
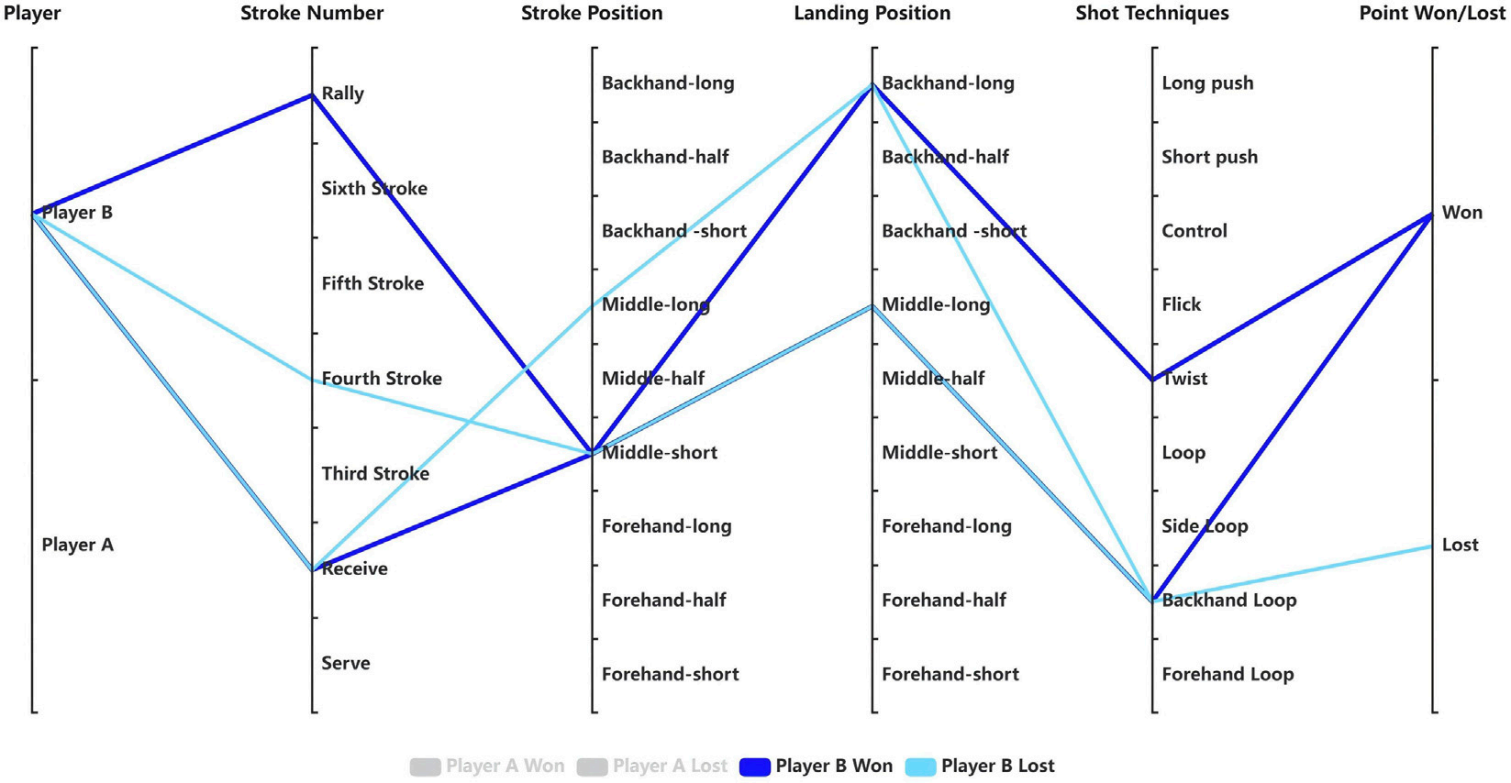
Player B tactical profiles derived from K-Modes aggregation.

Figures 6, 7 illustrate the clustered technical and tactical data for Player A and Player B, respectively, focusing on individual-level analysis. The technical and tactical characteristics of Player A in this match can be derived from Figure 6, while those of Player B are revealed in Figure 7. This interactive parallel coordinates visualization method facilitates the analysis of individual players by allowing users to selectively observe the required information, thereby enabling the rapid analysis. The player-specific visualization further reveals differences in tactical tendencies between Player A and Player B. For Player A, the winning profiles are more closely related to serve and third-stroke sequences. The representative winning paths show that Player A’s successful points were associated with serve or third-stroke actions, forehand-short or middle-short stroke positions, backhand-long or middle-long landing positions, and attacking techniques such as forehand loop and side loop. This indicates that Player A was more likely to obtain favorable outcomes when the early stroke sequence created opportunities for proactive attack or placement control. However, Player A’s losing profiles appear more clearly in the rally stage and are associated with backhand-loop execution, suggesting that Player A’s technical and tactical effectiveness decreased when the point developed into longer rally sequences.

For Player B, the winning profiles are mainly associated with receive or rally-related sequences, middle-short or backhand-long stroke positions, backhand-long or middle-long landing positions, and active techniques such as twist and backhand loop. These profiles indicate that Player B’s successful patterns were more frequently characterized by extended rally structures, middle or backhand-oriented placement, and proactive technical execution. In contrast, Player B’s losing profiles are more closely related to transitional stroke sequences and backhand-oriented execution, suggesting that some backhand-side transitions were less stable in the analyzed match.

Overall, the cluster-enhanced parallel coordinates visualization provides clearer tactical evidence than the original event-level display. It not only reduces visual clutter and line occlusion, but also helps identify representative co-occurrence patterns among player name, stroke number, stroke position, landing position, shot technique, and point outcome. The results indicate that Player A’s advantage was more evident in serve and third-stroke patterns, whereas Player B showed stronger performance in receive and rally-related sequences. These findings demonstrate the practical value of K-Modes-based categorical aggregation for exploratory post-match technical and tactical diagnosis in table tennis.

### 4.2 Stroke-sequence-oriented spatiotemporal cube visualization

Traditional two-dimensional landing point distributions often suffer from severe visual occlusion (Wu et al., 2018), which obscures athletes’ stroke preferences and their tactical evolution throughout a rally. In this context, stroke preference is defined as an athlete’s systematic tendency to use specific techniques or target particular zones under specific match conditions. The comprehensive analysis of these preferences—particularly their dynamic transitions across successive strokes—is essential for deconstructing an athlete’s tactical framework, predicting match trajectories, and formulating targeted competitive strategies (Wu et al., 2022).

The stroke-sequence-oriented spatiotemporal cube method aims to map shot landing distributions into a three-dimensional analytical space, where the sequential dimension is discretized by stroke number rather than generic clock time. To mitigate the visual clutter inherent in three-dimensional representations of spatiotemporal stroke data, the framework follows the original pipeline of data initialization and preprocessing, trajectory segmentation, sub-trajectory clustering, and multidimensional rendering.

The innovation of this module lies in adapting the general Space-Time Cube principle to the technical and tactical logic of table tennis. Specifically, the temporal axis is replaced by stroke-sequence progression, each rally is modeled as a discrete landing-point sequence, and sub-trajectory clustering is used to extract recurring landing-path patterns and hotspot evolution across successive strokes.

#### 4.2.1 Spatiotemporal cube model

**FIGURE 8:**
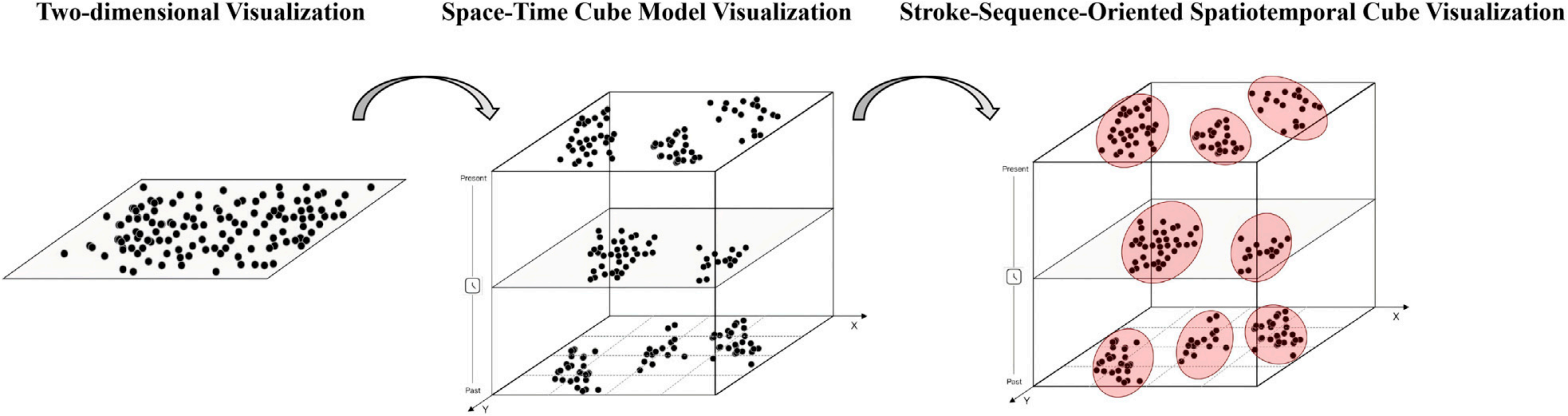
Stroke-sequence-oriented spatiotemporal cube visualization.

The Space-Time Cube Model represents the evolution of a two-dimensional plane over time using a three-dimensional solid figure, where each time point corresponds to a state on the two-dimensional plane (Bach et al., 2014). As shown in Figure 8, the height of the cube represents the time of event occurrence, while its base indicates the spatial location at the time of event. In the stroke-sequence-oriented spatiotemporal cube, the X-Y plane represents the table tennis table, and the Z-axis represents the number of stroke. The visualization targets clustered “hotspot regions” rather than raw scatter points. Each cluster forms a “hotspot column” in 3D space, where its planar position represents landing patterns and its Z-axis height indicates the active stroke number stage for that pattern (Andrienko and Andrienko, 2013). This view provides an intuitive understanding of a player’s attack focus and tactical patterns across different stroke numbers.

Each landing point in a rally can be represented as a spatiotemporal point:

$$p_{r,i}=\left(x_{r,i},y_{r,i},s_{r,i}\right)$$

where p_r,i_ denotes the *i*th landing point in the *r*th rally, x_r,i_ and y_r,i_ denote the landing-point coordinates on the table, and s_r,i_ denotes the corresponding stroke number. This definition ensures that the vertical dimension reflects table tennis specific stroke progression. The visualization targets clustered hotspot regions rather than raw scatter points alone. Each cluster forms a hotspot structure in the three-dimensional space, where its planar position represents the dominant landing area, its vertical position indicates the active stroke-number stage, and its size or density reflects the frequency of landing points in that region.

#### 4.2.2 Data analysis

Each rally is treated as a discrete landing-point sequence, with each landing point representing a data point in the spatiotemporal cube. This definition is different from a continuous ball-flight trajectory: the available data describe the landing position of each stroke, not the full physical flight path of the ball. An algorithm based on the Minimum Description Length (MDL) principle is used to extract feature points and segment long landing-point sequences into meaningful sub-trajectory segments.

Definition 1 Landing-point sequence dataset. Let the dataset be denoted as:

$$T=\left\{T_{1},T_{2},\ldots ,T_{R}\right\}$$

where R denotes the number of rally sequences. The r -th rally sequence is represented as:

$$T_{r}=\langle p_{r,1},p_{r,2},\ldots ,p_{r,n_{r}}\rangle$$

where $n_{r}$ denotes the number of valid landing points in the r -th rally. Each point $p_{r,i}$ takes the form defined in Section 4.2.1 and may also be associated with additional technical or tactical attributes, such as player name, stroke number, and shot technique.

Definition 2 Feature point dataset. Each rally sequence $T_{r}$ is divided into a smaller ordered feature point set:

$$CHT_{r}=\langle p_{r,1}^{ch},p_{r,2}^{ch},\ldots ,p_{r,c_{r}}^{ch}\rangle$$

where $c_{r}$ denotes the number of feature points extracted from the r -th rally sequence. The feature points preserve the main structural changes of the landing-point sequence and provide the basis for sub-trajectory segmentation.

Definition 3 Sub-trajectory segment dataset. Sub-trajectory segments are generated from adjacent feature points:

$$L_{r,h}=\bar{p_{r,h}^{ch}p_{r,h+1}^{ch}},h=1,2,\ldots ,c_{r}-1$$

where $L_{r,h}$ denotes the segment between the h -th and $\left(h+1\right)$ -th feature points in the r -th rally. The complete sub-trajectory segment set is then defined as:

$$S=\underset{r=1}{\overset{R}{⋃}}\left\{L_{r,1},L_{r,2},\ldots ,L_{r,c_{r}-1}\right\}$$

This construction allows the model to capture local sequential patterns, such as serve-to-third-stroke placement, receive-to-fourth-stroke transition, and landing-position shifts before entering the rally phase. Invalid or incomplete landing records are removed before segmentation, and the remaining sequences are checked to ensure that the original stroke order is preserved.

#### 4.2.3 Clustering algorithm

Density-based clustering is performed on the sub-trajectory segments using a metric that integrates perpendicular, parallel, and angular distances. This process identifies frequently occurring landing-path patterns with specific spatial orientations and positions during the competition. The original algorithmic logic is therefore retained, but the definitions of the trajectory object, distance metric, and clustering parameters are clarified to improve reproducibility.

For two landing-point sub-trajectory segments $L_{i}$ and $L_{j}$ , the integrated distance is defined as:

$$D\left(L_{i},L_{j}\right)=\alpha d_{⊥}\left(L_{i},L_{j}\right)+\beta d_{∥}\left(L_{i},L_{j}\right)+\gamma d_{\theta }\left(L_{i},L_{j}\right)$$

In this expression, the three distance components are interpreted as follows:

$d_{⊥}\left(L_{i},L_{j}\right)$ denotes the perpendicular distance between two segments.

$d_{∥}\left(L_{i},L_{j}\right)$ denotes the parallel displacement between two segments.

$d_{\theta }\left(L_{i},L_{j}\right)$ denotes the angular difference between two segments.

Before calculating the integrated distance, the perpendicular distance, parallel distance, and angular distance were normalized to a comparable scale. Since the purpose of the sub-trajectory clustering was to identify recurring landing-path transitions without imposing prior assumptions about the relative importance of spatial offset, longitudinal displacement, and directional change, equal weights were assigned to the three distance components. Specifically, the weights were set as α = β = γ = 1/3. Therefore, the integrated distance was calculated as the average contribution of the normalized perpendicular, parallel, and angular distances.

The neighborhood of a sub-trajectory segment $L_{i}$ is defined as:

$$N_{\varepsilon }\left(L_{i}\right)=\left\{L_{j}\in S|D\left(L_{i},L_{j}\right)\leq \varepsilon \right\}$$

where ε is the neighborhood distance threshold. Let minLns denote the minimum segment count, corresponding to the parameter minLns. If the number of neighboring segments satisfies:

$$\left|N_{\varepsilon }\left(L_{i}\right)\right|\geq \text{minLns}$$

then $L_{i}$ is regarded as a core segment and can be used to expand a cluster. Segments that do not satisfy this density condition and cannot be assigned to any cluster are treated as noise. In the final implementation, the neighborhood distance threshold was set as ε = 0.199, and the minimum segment count was set as minLns = 5. The parameter ε controls the maximum normalized distance within which two landing-point sub-trajectory segments are considered similar, while minLns defines the minimum number of neighboring segments required for a sub-trajectory segment to be regarded as a core segment. This parameter combination was selected by considering cluster stability, the proportion of noise segments, and the tactical interpretability of the resulting landing-path patterns. A smaller ε produced fragmented clusters with more noise segments, whereas a larger ε tended to merge tactically different landing transitions. The final setting preserved representative recurring landing-path patterns while avoiding excessive fragmentation or over-aggregation. The specific procedure for feature point extraction from landing-point sequence data is detailed in Algorithm 2.

Algorithm 2 **Landing-point sequence feature point extraction.**

 Input: A landing-point sequence $T_{r}$ .
 Output: The ordered feature point set $CHT_{r}$ .
 Begin.
  1. Add the first landing point of the rally to the feature point set.
  2. Initialize the start pointer and the segment length.
  3. Move the current pointer along the landing-point sequence.
  4. Calculate the partitioned and non-partitioned MDL costs for the segment from the current start point to the current point.
  5. If the partitioned cost is larger than the non-partitioned cost, add the previous landing point to the feature point set, update the start pointer, and reset the segment length.
  6. Otherwise, increase the segment length and continue scanning the sequence.
  7. Repeat Steps three to six until the endpoint of the rally sequence is reached.
  8. Add the final landing point of the rally to the feature point set.
 End.

Feature point extraction divides each landing-point sequence into multiple sub-trajectory segments. Sub-trajectory segments with high similarity are then aggregated to form representative landing-path patterns. The detailed procedure for sub-trajectory segment aggregation is summarized in Algorithm 3.

Algorithm 3 **Sub-trajectory segment aggregation algorithm.**

 Input: Segment set S , neighborhood distance threshold ε , and minimum segment count minLns.
 Output: Clustering result set C .
 Begin.
  1. Initialize the cluster index and mark all sub-trajectory segments as unclassified.
  2. For each segment $L_{i}$ in S , calculate its neighborhood $N_{\varepsilon }\left(L_{i}\right)$ .
  3. If $\left|N_{\varepsilon }\left(L_{i}\right)\right|<\text{minLns}$ , mark $L_{i}$ as noise.
  4. Otherwise, create a new cluster and assign $L_{i}$ and its neighboring segments to this cluster.
  5. Add the neighboring segments to the expansion queue.
  6. For each segment in the expansion queue, calculate its neighborhood.
  7. If the neighborhood size satisfies $\left|N_{\varepsilon }\left(L_{i}\right)\right|\geq \text{minLns}$ , assign the unclassified or noise segments in this neighborhood to the current cluster.
  8. Continue cluster expansion until no additional segments can be added.
  9. Repeat the process until all sub-trajectory segments have been processed.
  10. Output the clustering result set C .
 End.

#### 4.2.4 Analysis of table tennis spatiotemporal cube visualization results

To validate the stroke-sequence-oriented spatiotemporal cube module, Match Case 2 was used as the landing-point sequence analysis case. This match was selected from the final of another high-level international men’s table tennis event held in 2026, ended with a final score of four to two, and contained 109 rallies. A total of 487 valid landing-point records were collected from this match. An example of the landing-point records used for spatiotemporal cube visualization is shown in Table 2. The reliability of coordinate annotation was assessed using the intraclass correlation coefficient (ICC). The ICC values were 0.998 for the X-coordinate annotation and 0.996 for the Y-coordinate annotation, indicating excellent consistency in landing-point coordinate coding. The landing-point data were then visualized using the stroke-sequence-oriented spatiotemporal cube. In conventional two-dimensional landing-point views, landing positions from different stroke numbers are projected onto the same table plane. As a result, landing points from different rally stages may overlap substantially, which hinders the identification of stroke preferences and the analysis of tactical evolution across rally progression.

**TABLE 2:** Example of landing-point records used for spatiotemporal cube visualization.

Round	Score		Stroke number	Landing position X	Landing position Y
1	0	0	3	561	188
1	0	0	4	−1349	−183
.	.	.	​	​	.
5	10	11	5	−703	756
5	10	11	6	1271	−576

**FIGURE 9:**
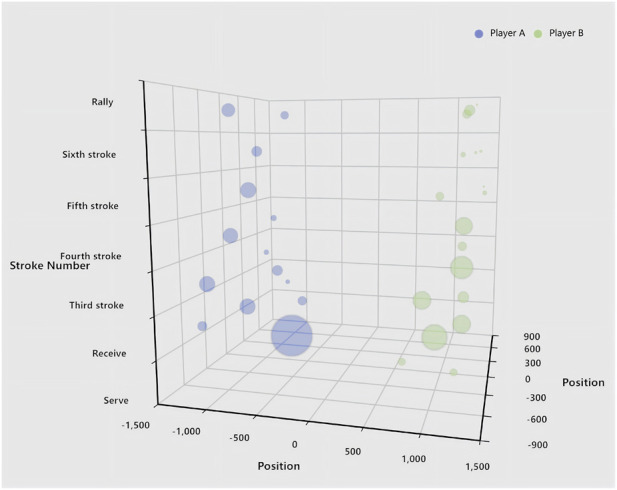
Stroke-sequence-oriented spatiotemporal cube visualization of landing-point hotspots.

In contrast, the stroke-sequence-oriented spatiotemporal cube provides a clearer analytical perspective by stratifying landing-point hotspots according to stroke number. As shown in Figure 9, the horizontal plane represents landing-point coordinates, the vertical axis represents stroke-sequence progression, and the bubble size indicates the relative concentration of landing points in the corresponding spatial region and stroke-number stage. The visualization shows that the two players had different hotspot distributions across stroke sequences, suggesting player-specific spatial preferences and changes in landing distribution during rally development. In particular, Player A, a left-handed player, showed relatively concentrated hotspots in several early- and middle-stroke stages, indicating a tendency to use placement variation after the serve-receive phase. Player B’s hotspots were more clearly distributed in several corresponding deep or middle areas across successive stroke stages, reflecting a different pattern of landing-point control during rally continuation.

**FIGURE 10:**
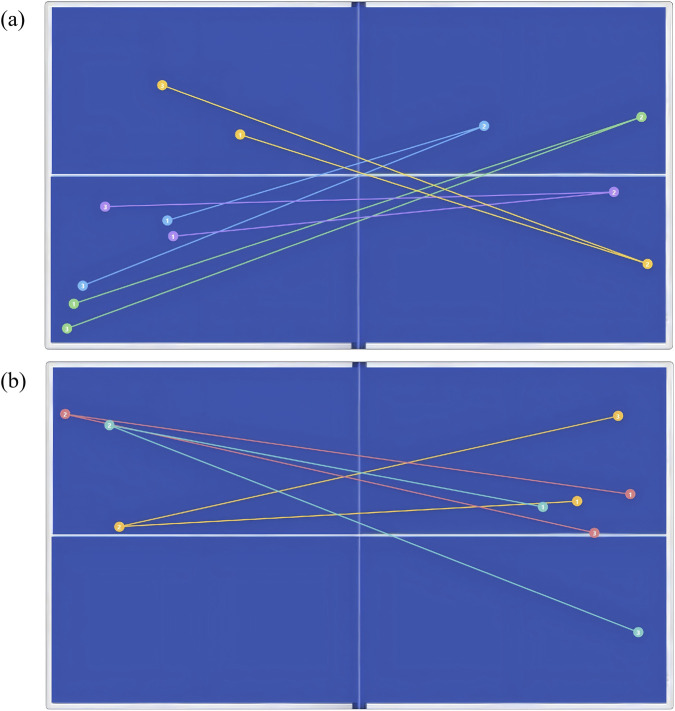
Sub-trajectory clustering results of landing-point sequences. **(a)** Representative sub-trajectory clustering result for Player A. **(b)** Representative sub-trajectory clustering result for Player B. Different colors indicate different representative sub-trajectory clusters, and the numbered nodes indicate the ball-order sequence within each clustered landing-path transition.

To further reveal how landing positions were sequentially connected across strokes, sub-trajectory clustering was conducted on the extracted landing-point transition segments. The results are shown in Figure 10. In the sub-trajectory clustering views, each colored polyline represents one representative landing-path transition cluster. The numbered nodes (1, 2, and 3) indicate the ball-order sequence within each clustered landing-path transition, rather than cluster labels or fixed stroke-number categories. For interpreting the directional characteristics of the clustered transitions, Player A is noted as a left-handed player. Thus, Figure 10 complements the hotspot visualization in Figure 9 by showing not only where landing points were concentrated, but also how these landing positions were connected across successive balls.

As shown in Figure 10a, Player A exhibited several repeated transition patterns. For backhand-side middle-short balls, Player A tended to return the ball either to the opponent’s forehand-side middle-short area or to the opponent’s backhand-side middle-long area. The opponent’s subsequent responses to these placements were generally directed back to Player A’s backhand-side middle-long area. For forehand-side middle-short balls, Player A tended to direct the ball to the opponent’s backhand-long area, after which the opponent often returned the ball to Player A’s forehand-long area. When handling balls from the backhand-long position, Player A generally redirected the ball to the opponent’s forehand-long area, while the opponent tended to press the following shot back toward Player A’s backhand-long area.

As shown in Figure 10b, Player B also showed repeated landing-path transition patterns. For short and long balls in the middle-to-forehand area, Player B generally directed the ball to Player A’s forehand-long area, after which Player A’s responses were distributed toward Player B’s backhand-long area or middle-long area. For half-long balls in the middle-to-forehand area, Player B tended to return the ball to Player A’s middle-long area, after which Player A continued to press the ball toward Player B’s forehand-long area. These clustered transitions suggest that the proposed sub-trajectory clustering method can reveal recurrent tactical routines, including short-to-deep transitions, cross-court pressure, and repeated targeting of long forehand or backhand areas.

The sub-trajectory clustering results further support the identification of recurring landing-path patterns. By grouping similar landing-point transitions, the visualization highlights repeated spatial routines embedded in rally progression. These clustered sub-trajectories help analysts examine how players construct tactical pressure through sequential placement rather than through isolated landing points.

In summary, by combining stroke-sequence-based spatiotemporal representation with sub-trajectory clustering, the proposed module extends traditional two-dimensional landing-point visualization. It reduces the visual clutter caused by overlapping landing points, clarifies the evolution of landing hotspots across stroke numbers, and provides an interpretable representation of recurring landing-path patterns. This module therefore offers complementary evidence for professional post-match technical and tactical analysis.

### 4.3 System implementation and integration

To operationalize the proposed analytical methods, we developed a specialized Table Tennis Match Visual Analytics System. Designed to support coaches and analysts in post-match technical and tactical diagnosis, the system provides a comprehensive pipeline encompassing data ingestion, preprocessing, and multimodal visualization rendering.

**FIGURE 11:**
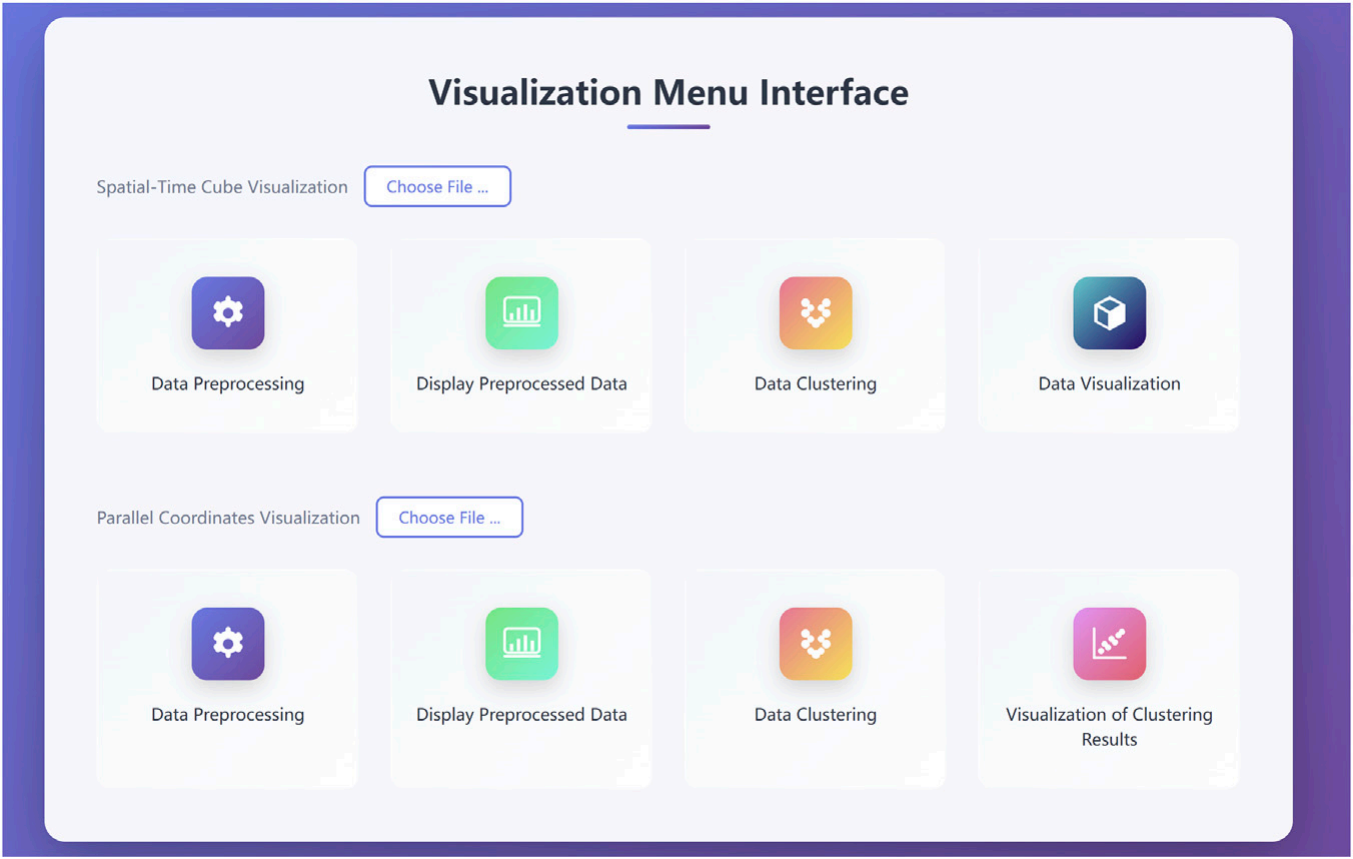
Visualization menu interface.

To ensure high modularity and scalability, the proposed system is architected based on the MVC design pattern. The backend employs a MySQL database for robust structured data management, while the frontend leverages the ECharts library to achieve high-performance, interactive visualization rendering. The platform integrates three core functional modules: (1) a Descriptive Statistics Module for quantifying fundamental technical and tactical indicators; (2) a Cluster-Enhanced Parallel Coordinates Module based on K-Modes aggregation for exploring representative multidimensional categorical stroke-event patterns; and (3) a Stroke-Sequence-Oriented Spatiotemporal Cube Module for modeling landing-point evolution and recurring landing-path patterns across stroke sequences. Furthermore, by implementing interactive view linkage (cross-view coordination) technology, the system synchronizes multiple analytical perspectives, thereby facilitating multidimensional tactical exploration for users. Figure 11 depicts the visualization menu interface of the system, showcasing the various analytical modules available to the user.

### 4.4 System application and expert feedback

To further evaluate the usability and analytical value of the proposed visual analytics system, an expert evaluation was conducted in response to the reviewer’s suggestion. Five experts were invited to participate in the evaluation. Among them, three experts had more than 10 years of professional experience in table tennis technical and tactical analysis, and two experts had backgrounds in computer science with published research related to table tennis analysis. The evaluation adopted a seven-point Likert scale, ranging from 1 = strongly disagree to 7 = strongly agree.

The questionnaire included 20 items covering five dimensions: readability of visualization, tactical interpretability, consistency with raw match records, practical usefulness, and overall evaluation. The experts evaluated the system after viewing the visualization results generated from the two match cases. Since the purpose of this evaluation was to obtain structured expert judgments on the proposed visual analytics framework, only Likert-scale scoring was used.

The expert evaluation results are shown in Table 3. Overall, the system received positive ratings from the five experts. The mean score across all items was 6.06 ± 0.63, indicating that the experts generally agreed with the effectiveness and usefulness of the proposed framework. Among the five dimensions, readability of visualization received the highest mean score (6.30 ± 0.57), suggesting that the cluster-enhanced parallel coordinates and stroke-sequence-oriented spatiotemporal cube improved the visual presentation of multidimensional table tennis data. Tactical interpretability also received a high rating (6.08 ± 0.70), indicating that the system was considered helpful for identifying scoring and losing patterns, comparing player-specific tactical differences, and interpreting the relationships among stroke sequence, shot technique, landing position, and point outcome.

**TABLE 3:** Expert evaluation results based on a seven-point Likert scale.

Evaluation dimension	Number of items	Mean ± SD
Readability of visualization	4	6.30 ± 0.57
Tactical interpretability	5	6.08 ± 0.70
Consistency with raw match records	4	5.90 ± 0.72
Practical usefulness	4	6.05 ± 0.51
Overall evaluation	3	5.93 ± 0.59
Total	20	6.06 ± 0.63

The practical usefulness dimension obtained a mean score of 6.05 ± 0.51, showing that the experts considered the system valuable for post-match tactical analysis, training preparation, and opponent analysis. The consistency with raw match records dimension obtained a mean score of 5.90 ± 0.72, suggesting that the visualized tactical patterns were generally consistent with the original match records, although clustering inevitably simplified some individual stroke-event details. The overall evaluation dimension obtained a mean score of 5.93 ± 0.59, further supporting the understandability and auxiliary diagnostic value of the proposed system.

It should be noted that this expert evaluation was conducted with a small group of domain and technical experts and should therefore be interpreted as preliminary evidence rather than a large-scale user study. Nevertheless, the results indicate that the proposed system provides a readable, interpretable, and practically useful visual analytics approach for exploratory post-match table tennis technical and tactical diagnosis.

## 5 Discussion

This study explored a multimodal visual analytics approach for table tennis technical and tactical analysis by integrating cluster-enhanced parallel coordinates with stroke-sequence-oriented spatiotemporal cube visualization. Rather than aiming to reproduce the original match data in a one-to-one manner, the proposed framework was designed to provide an abstract and interpretable representation of multidimensional stroke-event data and landing-point sequences. Through categorical pattern aggregation, stroke-sequence-based representation, sub-trajectory clustering, and interactive visual mapping, the system transforms complex annotated match records into visual patterns that reflect the dominant technical and tactical tendencies contained in the original data.

The cluster-enhanced parallel coordinates method provides an effective approach for summarizing multidimensional stroke-event data. Traditional parallel coordinates can display multiple tactical attributes simultaneously; however, when complex match data are directly visualized, severe line overlap and visual occlusion often reduce interpretability (Heinrich and Weiskopf, 2013). By introducing K-Modes clustering, the proposed method aggregates categorical stroke-event records into representative tactical profiles, which improves the readability of relationships among player name, stroke number, stroke position, landing position, shot technique, and point outcome. In this study, the clustered profiles should be understood as case-specific visual abstraction units rather than as universal tactical categories. Their value lies in helping analysts identify representative co-occurrence patterns among player name, stroke-event attributes, and point outcomes, thereby supporting exploratory interpretation of player-specific tactical tendencies.

The stroke-sequence-oriented spatiotemporal cube visualization complements the parallel coordinates module by emphasizing the dynamic evolution of landing-point distributions. In table tennis, tactical meaning is closely related to the sequential structure of rallies, including serve, receive, third stroke, fourth stroke, fifth stroke, and rally phase. Therefore, using stroke number as the sequential dimension is more consistent with the tactical logic of table tennis than relying solely on continuous clock time. By representing each rally as a discrete landing-point sequence and applying sub-trajectory clustering, this module transforms overlapping two-dimensional landing-point distributions into structured spatiotemporal hotspot patterns and recurring landing-path representations. Although this process inevitably involves data abstraction and aggregation, the resulting visualization can still reflect the main spatial tendencies and sequential changes observed in the original annotated data.

Compared with traditional notational analysis and static statistical charts (Hughes and Bartlett, 2002), the proposed framework offers a more integrated and process-oriented approach to technical and tactical interpretation. Conventional methods often focus on isolated indicators, such as stroke frequency, landing zones, or point outcomes, but they are less effective in revealing relationships among multiple tactical variables. In contrast, the proposed framework combines multidimensional categorical-event visualization with stroke-sequence-oriented spatiotemporal visualization, allowing tactical behavior to be examined from both attribute-based and sequence-based perspectives (Perin et al., 2018). This domain-specific design is particularly relevant for table tennis, where rapid stroke transitions and spatial placement changes play an important role in shaping match outcomes.

The structured expert evaluation further supports the readability and practical value of the proposed visual analytics system. In response to the need for more systematic validation, five experts were invited to evaluate the system using a seven-point Likert scale. Among them, three experts had more than 10 years of professional experience in table tennis technical and tactical analysis, and two experts had computer science backgrounds with published research related to table tennis analysis. The evaluation covered five dimensions: readability of visualization, tactical interpretability, consistency with raw match records, practical usefulness, and overall evaluation. The overall mean score across all items was 6.06 ± 0.63, indicating that the experts generally agreed with the usefulness and interpretability of the proposed framework. In particular, the high rating for readability of visualization suggests that the cluster-enhanced parallel coordinates and stroke-sequence-oriented spatiotemporal cube can help reduce perceptual complexity and support the interpretation of multidimensional tactical information.

Several limitations should be acknowledged. First, because the framework relies on clustering and visual abstraction, some detailed information from individual stroke events may be simplified during the visualization process. Therefore, the results should be interpreted as a structured representation of major tactical tendencies rather than as a complete replacement for raw data analysis. Second, the clustering results may be influenced by parameter settings, including the number of clusters in K-Modes aggregation and the distance threshold and minimum segment count used in sub-trajectory clustering. Although the present study supplemented the K-value selection process and clarified the sub-trajectory clustering procedure, future studies should further examine parameter sensitivity and interactive parameter adjustment. Third, the current validation was based on two elite match cases, each serving a specific analytical module. Although the categorical annotations and landing-point coordinates were checked for reliability and the expert evaluation provided preliminary support, the generalizability of the findings should be further validated using larger datasets across different players, match types, and competition levels. Fourth, the current dataset mainly includes player name, stroke number, stroke position, landing position, landing coordinates, shot technique, and point outcome, while other important tactical variables, such as spin, stroke speed, ball trajectory height, and player movement, were not fully incorporated. Including these variables may further improve the depth and tactical interpretability of the visualization results.

Overall, this study should be regarded as an exploratory attempt to apply visual analytics methods to table tennis spatiotemporal tactical data. The proposed framework does not aim to replace detailed statistical analysis or expert judgment, but rather to provide an intuitive and structured visual representation that helps analysts understand dominant tactical patterns and landing-sequence evolution within complex match data. Future research should validate the framework using larger datasets, involve more experts and coaches in systematic evaluation, and examine its applicability to other racket sports such as badminton and tennis.

## 6 Conclusion

This study proposed a multimodal visual analytics framework for exploratory post-match technical and tactical diagnosis in table tennis. The framework integrates cluster-enhanced parallel coordinates, stroke-sequence-oriented spatiotemporal cube visualization, and an interactive system implementation.

The K-Modes-based parallel coordinates module aggregates categorical stroke-event records into representative tactical profiles, helping reduce visual clutter and reveal co-occurrence patterns among player name, stroke number, stroke position, landing position, shot technique, and point outcome. The spatiotemporal cube module organizes landing-point data according to stroke-sequence progression and supports the observation of landing-distribution evolution and recurring landing-path patterns.

Using two elite match cases for module-specific validation, the framework demonstrated its potential for interpreting multidimensional tactical patterns and stroke-sequence-based spatial evolution. The reliability checks of categorical annotations and landing-point coordinates, together with the structured expert evaluation, further supported the readability, interpretability, and practical value of the proposed system.

Overall, this study provides a domain-adapted visual analytics approach for table tennis tactical analysis. Future work will expand the dataset, incorporate richer technical and contextual variables, and conduct broader user evaluations to further validate the framework.

## Data Availability

The raw data supporting the conclusions of this article will be made available by the authors, without undue reservation.
